# Examining the Role of Aging Education: Educational Themes Across 2013-2019 GSA Conference Presentations

**DOI:** 10.1093/geroni/igaa057.040

**Published:** 2020-12-16

**Authors:** Lisa Hollis-Sawyer

**Affiliations:** Northeastern Illinois University, Chicago, Illinois, United States

## Abstract

Gerontology education and the goal of “geriatric competence” are considered invaluable within the field but barriers exist in communicating ideas and training needs across areas of specialization. The main challenge of higher education institutions throughout the world is to develop professionals capable of understanding and responding to the current issues of diverse aging populations. The specific focus of the presentation will be the examination of aging education “themes” within and across different conference sections’ presentations in GSA conference proceedings across the last six years. Specifically, this presentation will review the outcomes of qualitative content analyses from several GSA conferences’ research presentations regarding the role of education across different disciplines in the gerontology field. Thematic analysis of several past years GSA conference programs of 2013 through 2019 yielded some of the following education-related themes (sample): theme #1: community education for clients, practitioners and applied service providers, theme #2: intergenerational educational mentoring/programming among researchers and practitioners in the field, and theme #3: professional knowledge updating for geriatric professionals. The implications for increasing emphasis on ongoing geriatric education for all professionals in the field and those they serve (older adults, families, and community members) will be discussed. This presentation offers insights regarding trends in educational issues within the field as well as the unique valuation of gerontology education within different areas of field specialization. The importance of examining different areas of specialization exists because different aging-related disciplines can share knowledge and resources in providing an integrated array of educational and training options.

